# Improvement in gait pattern after total knee arthroplasty for knee osteoarthritis

**DOI:** 10.1002/jeo2.70103

**Published:** 2025-04-01

**Authors:** Yasushi Oshima, Tokifumi Majima

**Affiliations:** ^1^ Department of Orthopaedic Surgery Nippon Medical School Tokyo Japan

**Keywords:** abnormal body posture, gait balance, knee osteoarthritis, knee–hip–spine syndrome, total knee arthroplasty

## Abstract

**Purpose:**

Knee osteoarthritis (KOA) leads to gait and balance deficiency, and to hinder activities of daily living (ADLs); all these factors may increase the risk of falls. Total knee arthroplasty (TKA) improves gait and balance; however, the effect of the contralateral knee's condition on this improvement has not been examined. This study evaluated the variation in gait pattern following TKA in relation to the different KOA levels or post‐TKA conditions of the contralateral knee, examining the potential role of TKA in fall prevention.

**Methods:**

This prospective study included 53 post‐TKA patients at 1‐year follow‐up. The gait parameters of symmetry—namely gait ability, uniformity as an index of gait and balance, and steps to reflect the level of ADL—were measured using the application software installed on the smart phone. Repeated measures analysis of variance was used for statistical analysis. The gaits were compared between patients with cruciate‐retaining (CR) and posterior‐stabilized (PS) implants and those with different conditions of the contralateral knee.

**Results:**

The preoperative uniformity, symmetry and steps were 59.0 ± 12.2%, 60.9 ± 12.9% and 50.4 ± 8.7, respectively. These gait parameters significantly decreased to 56.5 ± 12.2%, 58.5 ± 12.8% and 47.9 ± 8.6, respectively, 3 weeks after TKA; however, they significantly improved to 66.2 ± 10.7%, 68.5 ± 11.1% and 53.8 ± 4.9 1 year after TKA, respectively (*p* < 0.05). The variations of these parameters were similar between patients with CR and PS. Preoperative symmetry and uniformity declined with the progression of contralateral KOA grades, even after TKA. In contrast, the preoperative steps did not decrease with the increasing severity of contralateral KOA grades.

**Conclusions:**

Gait parameters of KOA patients deteriorated with contralateral KOA and even after TKA over time. However, TKA could improve knee function, gait patterns and ADL, thereby potentially preventing falls.

**Level of Evidence:**

Level III.

**Abbreviations**
ADL: activity of daily living
FTA: femorotibial angle
HKA: hip‐knee‐ankle
KA: kinematic alignment
KL: Kellgren‐Lawrence
KOA: knee osteoarthritis
MA: mechanical alignment
OA: osteoarthritis
QOL: quality of life
ROM: range of motion
SD: standard deviation
SVA: sagittal vertebral axis
TKA: total knee arthroplasty

## INTRODUCTION

Osteoarthritis (OA) is a degenerative joint disease that causes joint pain, limited range of motion (ROM), decreased activity of daily living (ADL) and lower quality of life (QOL). Hip OA and knee OA (KOA) were estimated to have a worldwide prevalence of 303.1 million in 2017 [19], representing a 9.3% increase since 1990, with further increases likely. Hip OA and KOA are known risk factors for falls, which are positively correlated with the number of symptomatic OA joints (hip and knee); specifically, the risk increased by 53%, 74% and 85% with one, two and three to four affected joints, respectively, compared with asymptomatic hip/knee joints [5]. Moreover, hip OA and KOA cause abnormal body posture with increased anterior inclination of the body trunk and concomitant low back pain, which has been defined as hip–spine and knee–spine syndromes [15, 16, 24]. Abnormal posture is also a crucial contributor to falls, which can lead to critical situations such as bone fractures; brain trauma; and increased emergency room visits, hospitalizations, and impairments in ADL and QOL. These factors negatively affect morbidity and mortality, especially in older populations [1, 7, 23]. Globally, the number of people aged ≧60 years reached 1 billion in 2018, representing 13% of the total population. By 2022, this number increased to 1.1 billion, with the largest numbers reported in China (264.7 million), India (148.7 million), the United States (79.3 million), and Japan (44.4 million). It is expected to reach 2 billion by 2047 (21% of the total population) and 3.1 billion by 2100 (30% of the total population) [25]. Therefore, it is necessary to prevent falls to increase healthy life expectancy and save medical expenses in the current ageing society worldwide.

Total knee arthroplasty (TKA) is a successful surgical intervention for advanced and end‐stage KOA to relieve pain, increase knee ROM and improve ADL and QOL with sufficient longevity and survivorship of artificial prostheses [3]. Recently, KOA‐induced abnormal posture with an increased sagittal vertebral axis (SVA), which is one of the body balance indices, has been demonstrated to improve after TKA as knee–hip–spine syndrome [17, 18]. However, SVA remains a statical index of body posture, and dynamic indices are still necessary for TKA assessment. We hypothesized that if KOA‐related body posture can be improved with TKA, then the gait patterns can also be improved. This study evaluated gait pattern variations after TKA by comparing the contralateral knee with respect to radiographic KOA grades or post‐TKA status.

## PATIENTS AND METHODS

Patients with primary KOA who were scheduled to undergo primary TKA between 1 April 2020 and 31 March 2021, and could be followed up for 1 year were enroled in this study. We excluded patients with arthritis secondary to a different aetiology, such as rheumatoid arthritis, trauma or previous surgical interventions to the knee, complications of severe adult spinal deformity and hip OA, post‐operative status of the spine or hip, and central or peripheral nerve disorders, to focus on the relationship between KOA conditions and gait and balance. Patients with neurological and otorhinolaryngological pathologies were also excluded. Furthermore, patients who could not walk for three sessions of 22 s during the present study were excluded, as it took approximately 22 s to measure the gait patterns and three sessions were required to improve the accuracy of these measurements. The hospital's Institutional Review Board approved this study (no. R1‐05‐1133), and all the patients provided written informed consent prior to performing any study‐related activities.

All TKAs were performed by a single surgeon using a mechanical alignment (MA) procedure. Cruciate‐retaining (CR) and posterior‐stabilized (PS) implants (Triathlon; Stryker Inc., and Logic; Exactech) were selected appropriately according to the ROM of the preoperative knee, severity of alignment deformity, and condition of the posterior cruciate ligament. CR implants were used in cases in which the preoperative knee extension angle was >−15°, knee flexion angle was >120°, radiographical hip–knee–ankle angle was >−15°, and posterior cruciate ligament was relatively intact. PS implants were used in all other cases. Post‐operative rehabilitation included knee ROM exercises and gait training without specific limitations, starting on the first post‐operative day.

Gait patterns were evaluated preoperatively, 3 weeks post‐operatively and 1 year post‐operatively using the application InStride (Ortho MX Technologies Inc.), which was developed to calculate the three‐dimensional position of the whole‐body centre of mass [12, 21]. Patients were instructed to walk along a passageway at our institution as straight as possible at their usual speed for approximately 22 s without break. Then, the whole‐body centre of mass trajectory was calculated as the gait pattern using the accelerometer function of the smartphone, which was placed in a waist pouch worn by the patient. Gait measurements were performed thrice at 1‐min intervals, and the average values were used for the evaluation. Patients were allowed to use a cane during the evaluation, when required. Three categories were evaluated: uniformity (gait regularity of whole stride), which reflects gait ability; symmetry (body balance of right and left steps in gait), which reflects gait and body balance; and the number of steps (physical activity), which reflects the level of ADL. Variations in the number of patients who used a cane were measured. The variations in the three categories with TKA were then evaluated. Patients were divided into CR and PS groups, and these variations were compared between these groups.

The contralateral knee conditions of all patients were also categorized using the Kellgren‐Lawrence (KL) classification (Grades 1–4), except for patients who had already undergone contralateral knee (post‐TKA).

### Data analyses

All data are expressed as the mean ± standard deviation. All statistical analyses were performed using the statistical package SPSS version 29.0.2.0 (IBM Corp.), with *p* < 0.05 considered significant. Repeated measures analysis of variance and post hoc pairwise comparison (Bonferroni test) were used to compare gait patterns before, 3 weeks after, and 1 year after TKA in patients. The paired *t*‐test was used to compare the gait patterns before and 1 year after TKA in patients with different contralateral knee conditions.

## RESULTS

The study analyzed 53 patients, and the demographics of these patients are described in Table 1.

**Table 1:** Patient demographics and clinical characteristics.

Patients	53 (male: 10, female: 43)
Age	77.1 ± 6.3 (65–89) years old
Height	152.1 ± 8.4 (136.0–173.5) cm
Weight	59.6 ± 10.2 (43.2–88.2) kg
Body mass index	25.7 ± 3.5 (19.6–37.9) kg/m^2^
Preoperative knee OA grades	KL 3: 6
	KL 4: 47
OA deformity types	Varus deformity: 50
	Valgus deformity: 3
Contralateral knee conditions	KL‐1: 3
	KL‐2: 7
	KL‐3: 15
	KL‐4: 13
	Post‐TKA: 15

Abbreviations: KL, Kellgren‐Lawrence; OA, osteoarthritis; TKA, total knee arthroplasty.

**Figure 1:**
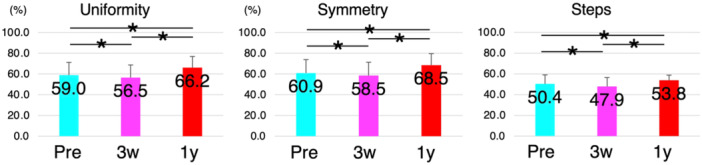
Variations of the uniformity, symmetry and steps for all patients with total knee arthroplasty (TKA). Of the 53 participants, uniformity, symmetry and steps were significantly decreased 3 weeks after TKA compared with the preoperative values. However, these parameters significantly increased 1 year after TKA. Pre, preoperatively; 3w, 3 weeks after TKA; 1y, 1 year after TKA.

Of the 53 patients, 33 (62.3%) used a cane before TKA. This number increased to 45 (84.9%) 3 weeks post‐operatively, and decreased to 16 (30.2%) 1 year post‐operatively. The uniformity, symmetry, and number of steps significantly decreased 3 weeks after TKA. However, the parameters improved 1 year after TKA and were significantly higher than their preoperative values (*p* < 0.05) (Figure 1).

**Figure 2:**
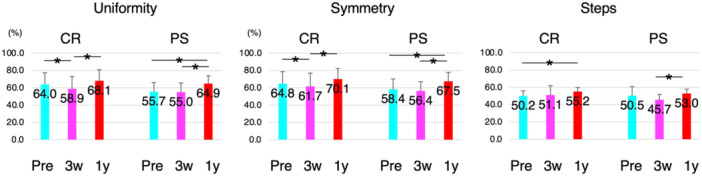
Variations of uniformity, symmetry, and steps among participants who underwent cruciate‐retaining (CR) or posterior‐stabilized (PS) total knee arthroplasty (TKA). In the case of CR TKA, uniformity and symmetry significantly decreased 3 weeks after TKA but significantly increased 1 year after TKA compared with preoperative values. The number of steps increased significantly 1 year after TKA compared with preoperative values. In PS TKA, uniformity and symmetry increased 1 year after TKA compared with preoperative and 3 weeks after TKA values. The number of steps also increased 1 year after TKA. Pre, preoperatively; 3w, 3 weeks after TKA; 1y, 1 year after TKA.

Of the 53 patients, 21 received the CR implant, and 32 received the PS implant. When comparing gait pattern variations between the CR and PS groups, preoperative uniformity and symmetry were higher in the CR group than in the PS group. Both decreased 3 weeks after TKA but improved 1 year after TKA. Similarly, step counts decreased 3 weeks post‐TKA and improved 1‐year post‐TKA in the PS group, whereas the CR group showed an increase in steps over time post‐TKA (Figure 2).

**Figure 3:**
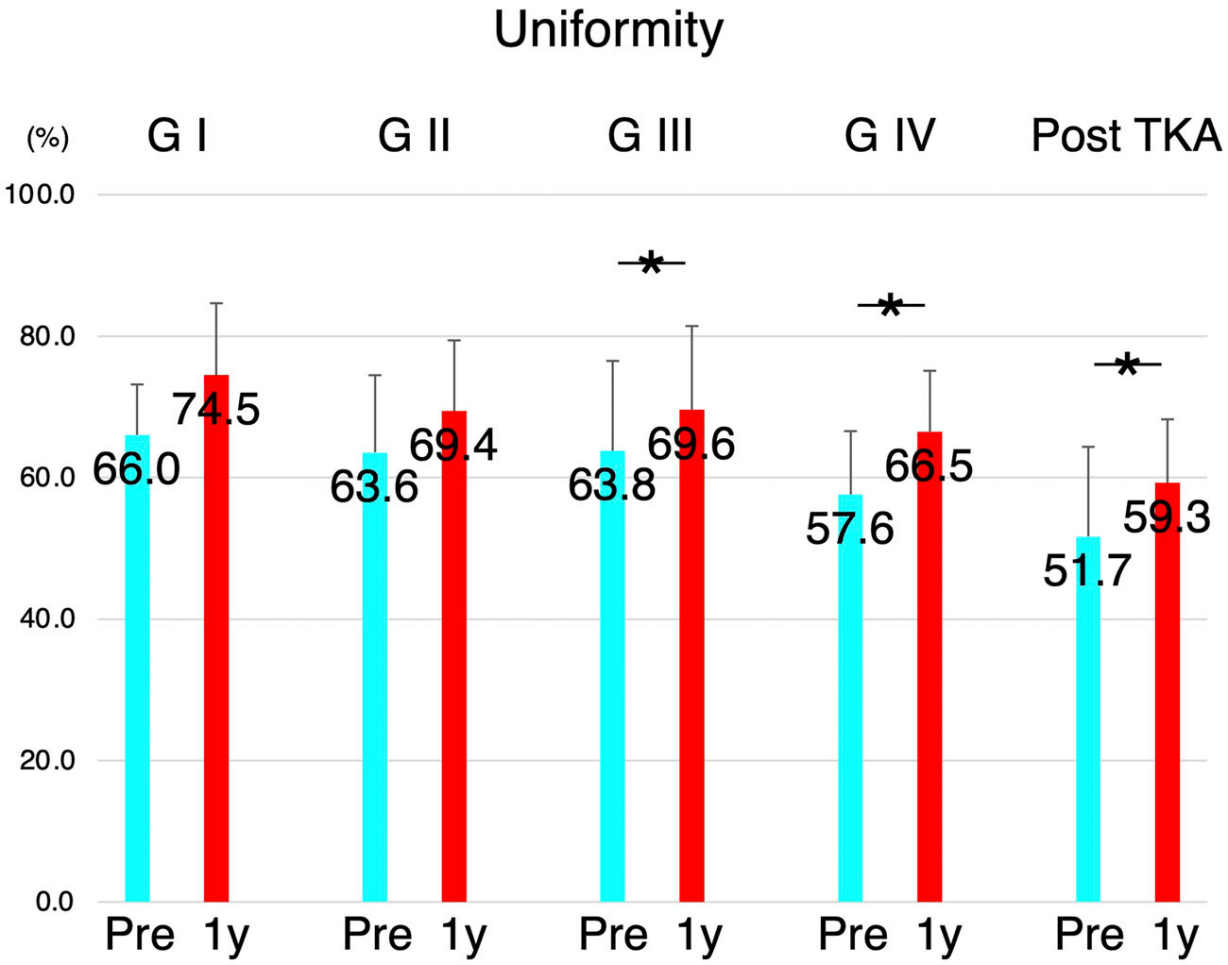
Variations of uniformity among all patients undergoing total knee arthroplasty (TKA). Participants were categorized according to the contralateral knee conditions. Preoperative uniformity was preserved in participants with lower osteoarthritis grades on the contralateral knee compared with those with severe osteoarthritis grades or uniformity after TKA. However, this parameter improved 1 year after surgery, regardless of contralateral knee conditions. G1, Kellgren‐Lawrence (KL) Grade 1 (*n* = 3); G2, KL Grade 2 (*n* = 7); G3, KL Grade 3 (*n* = 15); G4, KL Grade 4 (*n* = 13); Post‐TKA, TKA previously performed on the contralateral knee (*n* = 15); Pre, preoperatively; 1y, 1‐year post‐TKA.

**Figure 4:**
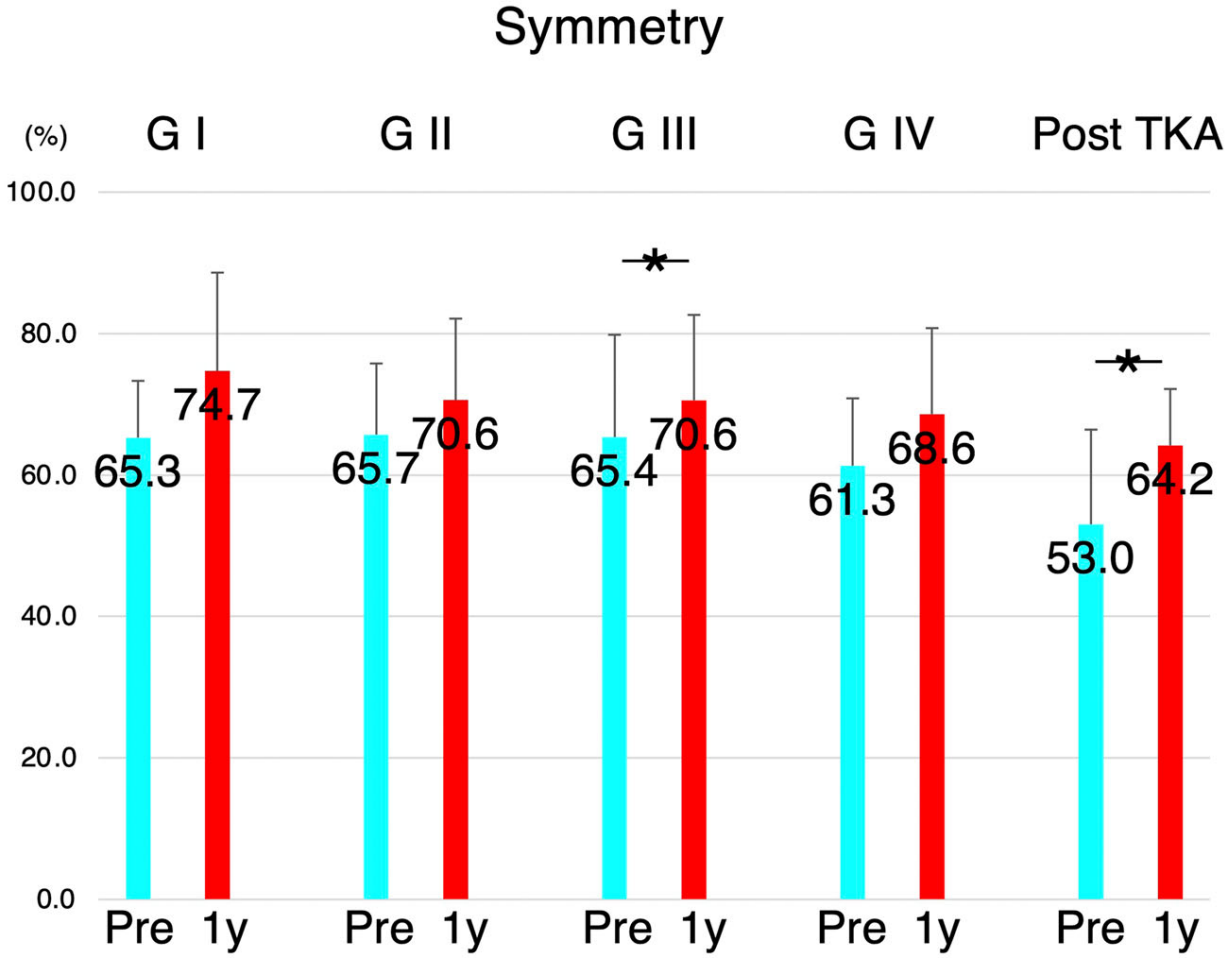
Variations of symmetry for all patients undergoing total knee arthroplasty (TKA). Preoperative symmetry was preserved in participants with lower osteoarthritis grades in the contralateral knee compared with those with Grade IV osteoarthritis or those with symmetry after TKA. However, this parameter improved 1 year after TKA, regardless of the contralateral knee conditions. G1, Kellgren‐Lawrence (KL) Grade 1 (*n* = 3); G2, KL Grade 2 (*n* = 7); G3, KL Grade 3 (*n* = 15); G4, KL Grade 4 (*n* = 13); Post‐TKA, TKA performed on the contralateral knee (*n* = 15); Pre, preoperatively; 1y, 1‐year post‐TKA.

**Figure 5:**
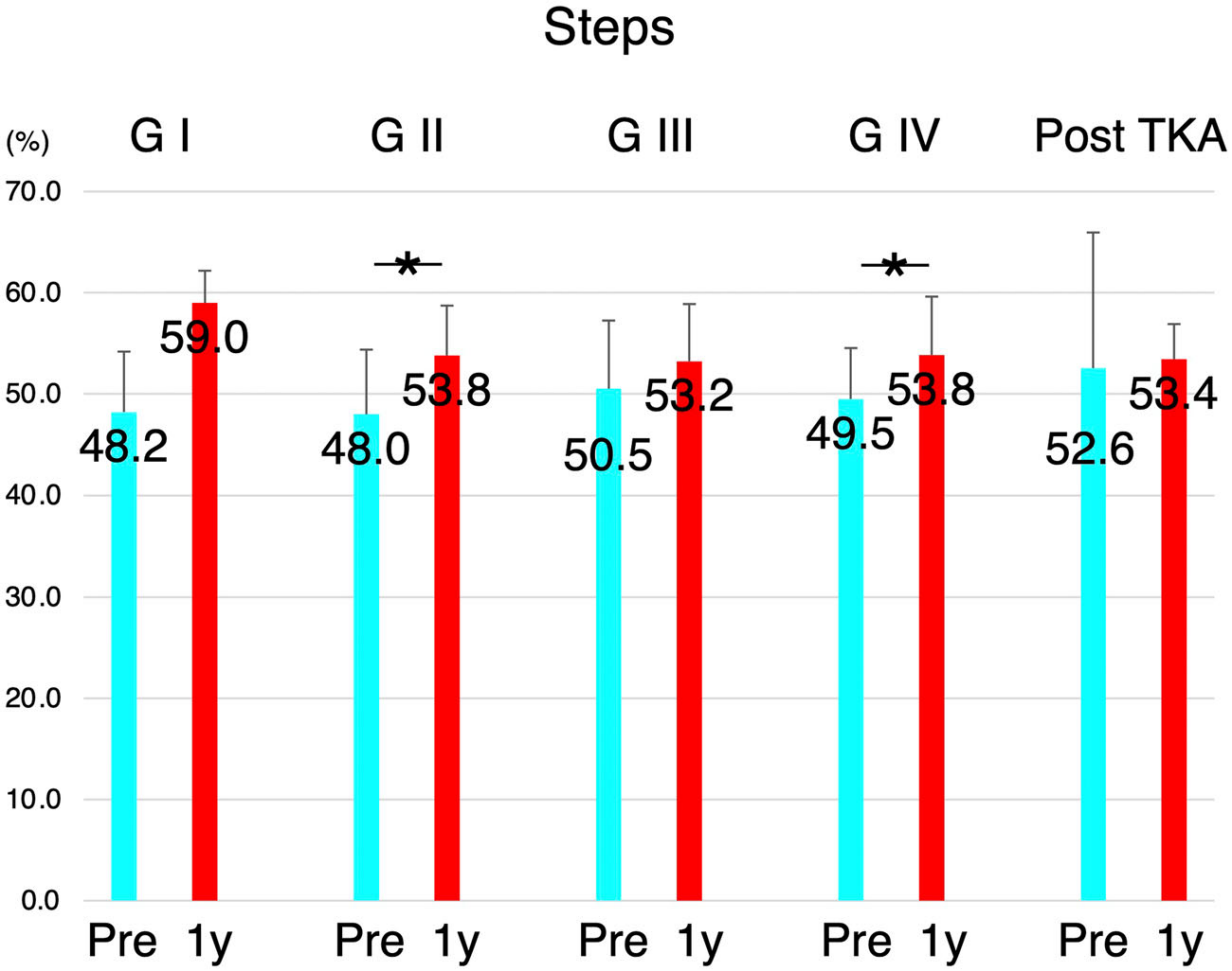
Variations of the steps for all patients undergoing total knee arthroplasty (TKA). The number of steps was similar in all groups regardless of preoperative contralateral knee conditions. For all groups, the values improved 1 year after TKA. G1, Kellgren‐Lawrence (KL) Grade 1 (*n* = 3); G2, KL Grade 2 (*n* = 7); G3, KL Grade 3 (*n* = 15); G4, KL Grade 4 (*n* = 13); Post‐TKA, TKA has been performed on the contralateral knee (*n* = 15), Pre: preoperatively, 1y: 1 year post‐operatively.

When comparing gait pattern variations based on different contralateral knee conditions, preoperative uniformity and symmetry were relatively preserved at lower OA grades but were even lower after the contralateral TKA condition. However, these parameters improved 1 year after TKA, regardless of the contralateral knee conditions (Figures 3 and 4). The number of steps was similar in all groups with different preoperative contralateral knee conditions, and this improved 1 year after TKA (Figure 5).

## DISCUSSION

Variations in gait patterns among patients with KOA who underwent TKA were evaluated in this study. Gait patterns deteriorated with increasing severity of the contralateral knee conditions, even after contralateral TKA. However, 1 year post‐operatively, gait patterns improved with TKA regardless of the contralateral knee condition.

The average life expectancy has been increasing globally. However, healthy life expectancy, defined as the number of years spent free of activity limitation at a certain age, is shorter than the average life expectancy. In Japan, the average life expectancy for men and women is 81.4 and 87.5 years, respectively; however, healthy life expectancy is 9 and 12 years shorter, respectively [14]. Thus, it is necessary to minimize these gaps in an ageing society, which can be achieved by fall prevention, as falls are a major cause of mortality and morbidity in older populations [6]. KOA and gait and balance impairments are major risk factors for falls, and abnormal body posture also results from KOA. Thus, proper treatments of KOA are considered to improve body balance and gait patterns, thereby preventing falls.

The SVA, which is the distance between the plumb line of the seventh cervical vertebra and the posterior–superior corner of the first sacral vertebra, has recently been identified as an index of sagittal body balance, which improves with TKA. Thus, KOA can cause abnormal body posture, previously known as knee–spine syndrome. However, because the abnormal body posture due to KOA improved with TKA, this condition has since been termed knee–hip–spine syndrome [17, 18]. In this study, the uniformity and symmetry of gait deteriorated 3 weeks post‐operatively, likely because the patients were still recovering from the surgical interventions. The recovery period of physical activity to reaching a plateau reportedly lasted 6 months after TKA [2]. Gait parameters significantly improved 1 year post‐operatively. Therefore, in addition to knee ROM and alignment, TKA can improve gait and balance deficits.

During the measurements, patients were asked to walk as straight as possible at their usual speed. The number of steps was similar in all patients with different contralateral knee conditions preoperatively, but this increased in all groups 1 year post‐operatively. As the number of steps is an index of physical activity, TKA can increase physical activity and the level of ADL. A higher activity level is generally considered to be associated with a lower fall risk of falls, but high‐activity individuals can also have an increased exposure to falls [4, 10]. Nevertheless, it has been demonstrated that higher physical activity can prevent falls after TKA [20]. Thus, apart from an increase in ADL, TKA may prevent falls in older populations.

Knee kinematics and gait patterns are usually evaluated using motion sensors under image intensifiers in a controlled setting, but this is a costly approach. In this study, a smartphone application to calculate the centre of mass of the whole body was used for measurements instead, in which knee kinematics was not evaluated; however, the gait patterns and physical activity were examined. Moreover, patients can use this application privately to improve the ADL. Thus, the proposed system is an easy and cost‐effective way to evaluate gait balance and steps.

A ‘one size fits all’ systematic approach, MA, has been developed to restore equal medial–lateral mechanical forces and to increase the longevity of the prostheses [9]. However, kinematic alignment (KA) was recently developed to achieve a personalized or patient‐specific alignment approach, and an improvement in knee function compared with the MA has been reported [8]. Long‐term patient satisfaction and clinical outcomes with KA do not surpass those achieved with MA [13]. Thus, the MA‐TKA was applied. However, CR and PS implants were selectively applied according to each patient's KOA condition at our institution. The crucial difference between these implants was that the posterior knee stability was preserved with native posterior cruciate ligament in the CR or was re‐established with the post‐cam mechanism of the TKA prosthesis in PS implants. Despite these characteristics, the gait patterns of these implant designs do not differ significantly [11, 22]. In the current study, gait patterns were similar between the groups. These findings suggest that TKA surgeons do not need to prioritize one approach over another when aiming to improve gait and balance after TKA.

The limitations of this study should also be discussed. The study had a small sample size and a follow‐up period of 1 year. Statistically significant improvements in gait patterns were observed, and the follow‐up period was longer than the 6‐month recovery period after TKA. However, further studies should include a large sample size with a longer follow‐up period to validate our results. Although uniformity and symmetry improved with TKA, the parameters of patients who underwent contralateral TKA were lower than those who did not. The duration between the previous and present TKAs was 29.7 ± 33.9 months. Therefore, after gait pattern improvement with TKA, it is suspected that the gait pattern deteriorates again after 1 year with time and ageing. Nevertheless, more data are needed to explore this issue.

## CONCLUSIONS

This study revealed that gait patterns and the level of ADL of KOA patients improve after TKA. Therefore, besides improving knee functions, TKA could resolve the critical issue of preventing falls in the ageing society.

## AUTHOR CONTRIBUTIONS

All authors participated in this study and read and approved the final manuscript. Yasushi Oshima set up the investigation, performed the operative procedures, followed up with the patients, analyzed the data and drafted the manuscript. Tokifumi Majima supervised the investigation, followed up with the patients and helped draft the manuscript.

## CONFLICT OF INTEREST STATEMENT

The authors declare no conflict of interest.

## ETHICS STATEMENT

The Institutional Review Board of Nippon Medical School approved this study (No. R1‐05‐1133). All patients provided informed consent and signed written consent forms.

## Data Availability

The aggregate data are included in the Results section. Please contact the corresponding author for raw data, including individual ratings.
